# Reference Power Optimization for Common-Path Probes in Optical Coherence Tomography

**DOI:** 10.3390/jimaging12090454

**Published:** 2026-09-20

**Authors:** Asha Parmar, Sora Alghziwatalkhawaldh, Shantanu Chauhan, Kanwarpal Singh

**Affiliations:** Department of Electrical and Computer Engineering, McMaster University, Hamilton, ON L8S 4L7, Canada; alghziws@mcmaster.ca (S.A.); shant8@mcmaster.ca (S.C.); singk33@mcmaster.ca (K.S.)

**Keywords:** optical coherence tomography, phase stability, power optimization, fiber-optic probe

## Abstract

Common-path optical coherence tomography (CP-OCT) has garnered significant interest for use in biomedical imaging due to its compact configuration, enhanced phase stability, and reduced sensitivity to environmental disturbances. The imaging performance of a common-path probe is significantly influenced by the reference power generated within the probe, which determines the interference modulation, signal-to-noise ratio (SNR), phase stability, and ultimately, the image quality. This study presents a systematic optimization of reference power in a fiber-based common-path OCT probe through numerical simulation and experimental validation. The optical coupling efficiency was evaluated using the Fiber Coupling analysis implemented in Zemax OpticStudio and validated experimentally using a fabricated probe incorporating an index-matching UV-curable adhesive as a partial Fresnel reflector. The influence of fiber-to-reflector separation on the coupled reference power was investigated and correlated with SNR, phase stability, and OCT image quality. The results demonstrate that increasing the reference power improves SNR and phase stability up to an optimum range of 20–60 µw, beyond which a gradual performance reduction is observed. In contrast, phase stability shows minimal additional improvement. The optimized reference power also enhances image contrast and structural visibility. These findings establish practical design guidelines for selecting the fiber-to-reflector spacing to achieve balanced reference power and improved imaging performance in compact common-path OCT probes for biomedical imaging applications.

## 1. Introduction

Optical coherence tomography (OCT) is a non-invasive imaging modality that provides high-resolution, depth-resolved visualization of biological tissues in real time. Owing to its ability to generate cross-sectional and three-dimensional images without tissue sectioning, OCT has become an indispensable tool in biomedical research and clinical practice, particularly in ophthalmology, dermatology, endoscopy, cardiovascular imaging, and tissue characterization [1,2,3,4,5,6]. Continuous advances in light sources, detectors, and signal processing have substantially improved imaging speed, sensitivity, and resolution, enabling OCT to address increasingly demanding biomedical and industrial imaging applications [1,3,7,8].

For example, high axial and lateral resolution are important for resolving fine retinal and epithelial structures in ophthalmic and dermatological imaging, whereas sufficient imaging depth, sensitivity, and probe compactness are particularly important for endoscopic and cardiovascular applications, where imaging is performed in scattering tissue using miniature probes. For phase-sensitive applications, such as optical coherence elastography, phase stability is particularly important because phase fluctuations can introduce errors in displacement measurements and, consequently, in the estimation of tissue mechanical properties [9].

Conventional OCT systems are generally based on a Michelson interferometer comprising separate reference and sample arms. Although this architecture provides high sensitivity and independent control of the reference signal, it requires precise optical alignment and can be sensitive to environmental disturbances, including vibration, temperature variations, and mechanical drift. These limitations become increasingly important in miniature, fiber-based, and handheld imaging systems, where maintaining stable alignment and optical path matching can be challenging. In contrast, CP-OCT uses a shared optical path for the reference and sample signals over a substantial portion of the optical system. Consequently, environmental perturbations that affect both signals similarly can be partially canceled, improving phase stability while reducing the number of optical components and simplifying probe integration. These characteristics make CP-OCT particularly attractive for compact, fiber-based probes intended for biomedical imaging applications [1,10,11,12,13,14].

In CP-OCT, the reference signal is typically generated by partial Fresnel reflection from an optical interface integrated into the probe, such as a fiber tip, coverslip, lens surface, or adhesive interface. Unlike conventional OCT systems, where the reference power can be independently adjusted, the reference power in CP-OCT is determined by the optical properties and geometry of the probe. Parameters including reflector reflectivity, fiber alignment, optical coupling efficiency, and fiber-to-reflector separation collectively influence the amount of reference light coupled back into the fiber and, consequently, the interference efficiency [15,16,17,18,19,20].

The reference power plays a critical role in determining OCT performance because the detected interference signal depends on the balance between the reference and sample fields. Insufficient reference power produces weak interference fringes, leading to reduced signal-to-noise ratio (SNR), increased phase fluctuations, and degraded image quality. Conversely, excessive reference power may limit the detector dynamic range and increase the contribution of intensity-related noise, resulting in reduced imaging performance. Therefore, selecting an appropriate reference power is essential for maximizing SNR, maintaining phase stability, and achieving high-quality OCT imaging [21,22,23]. Accordingly, reference-power optimization should not be evaluated solely in terms of optical power, but also based on its effect on key system-performance metrics, including SNR, phase stability, and OCT image quality.

Several studies have reported CP-OCT probes employing different reference-generating interfaces, including angle-polished fiber tips, partially reflective coatings, GRIN lenses, ball lenses, and integrated reflective elements. Previous investigations have demonstrated that reference signal generation significantly influences OCT sensitivity and imaging performance. However, most studies have focused on probe fabrication, optical design, or specific biomedical applications, while fewer have systematically investigated the relationship between probe geometry, optical coupling efficiency, reference power, SNR, phase stability, and image quality within a unified optimization framework. Such a quantitative framework is particularly relevant for compact probes, where optical alignment, reference power, detector dynamic range, and phase stability must be considered together to meet the performance requirements of different biomedical imaging applications [7,9,20,24,25,26,27,28,29].

In this study, we present a systematic optimization of reference power in a fiber-based CP-OCT probe through numerical simulation and experimental validation. Rather than focusing primarily on probe fabrication, optical configuration, or application-specific performance, this work establishes a quantitative relationship between fiber-to-reflector separation, optical coupling efficiency, and reference power, and evaluates its influence on SNR, phase stability, and OCT image quality. The optical coupling was simulated using Zemax OpticStudio and experimentally validated using a fabricated probe incorporating an index-matching UV-curable adhesive as a partial Fresnel reflector. The resulting analysis identifies a practical reference-power range that provides a favorable balance between interference strength and imaging performance, thereby establishing a simple, geometry-based strategy for optimizing compact CP-OCT probes.

## 2. Materials and Methods

### 2.1. Swept-Source Optical Coherence Tomography (SS-OCT) System

**Figure 1:**
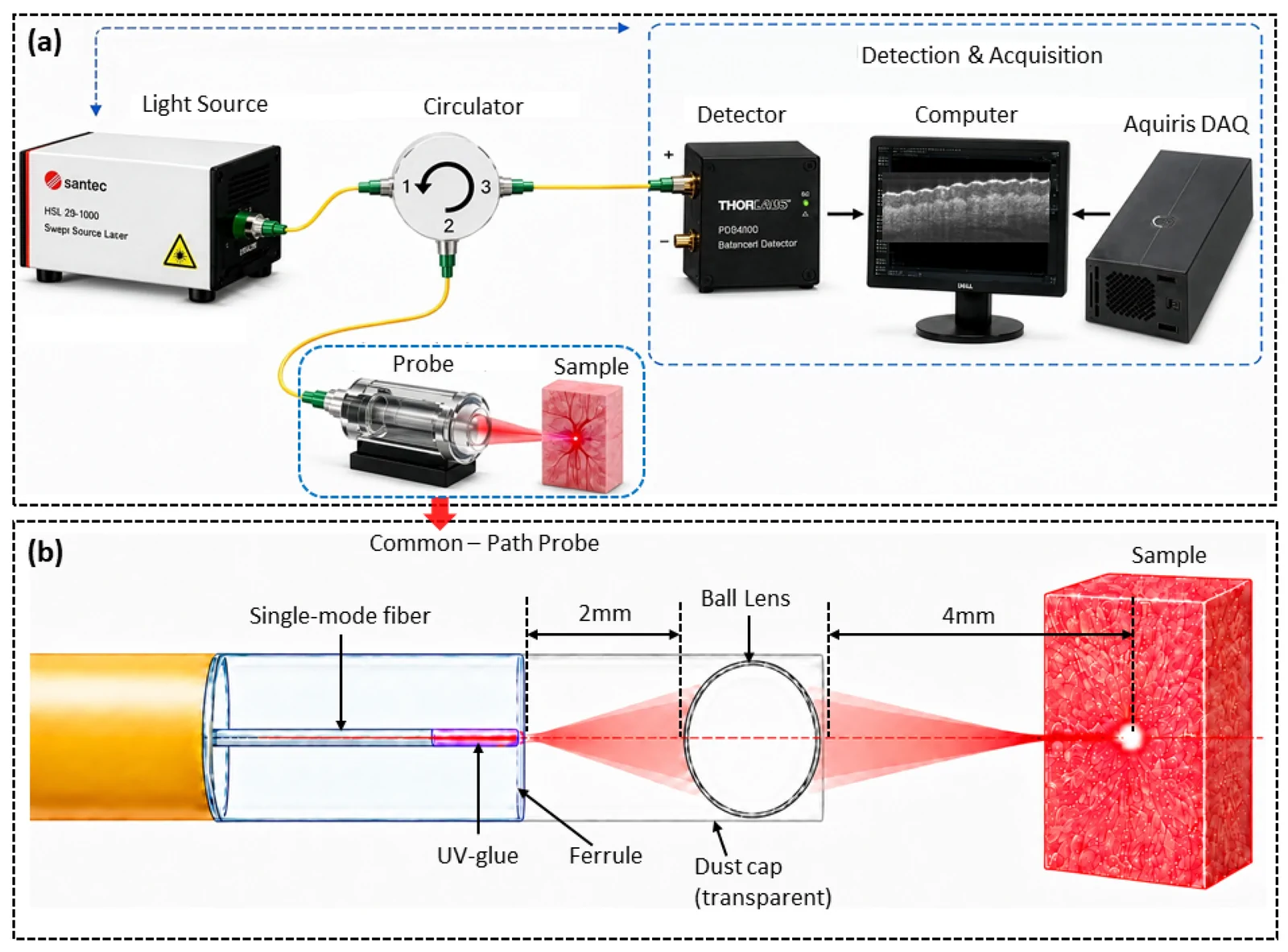
(**a**) Schematic of the common-path swept-source OCT system showing the light source, circulator, common-path probe, detection unit, and data acquisition module. (**b**) Schematic of the CP-OCT probe comprising a single-mode fiber, an index-matching UV-curable adhesive serving as a partial reflector, and a ball lens mounted inside a transparent tube. Arrows indicate signal/data flow; the red arrow indicates the expanded probe view.

A schematic of the SS-OCT system is shown in Figure 1a. The system employs a MEMS-based swept-source laser (HSL-20-100, Santec, Komaki, Japan) operating at a central wavelength of 1310 nm, with a spectral bandwidth of 105 nm, an output power of 18.7 mW, and a sweep rate of 100 kHz. The emitted light is directed through an optical circulator (CIR-1310-50-APC, Thorlabs, Newton, NJ, USA) to the common-path probe, with approximately 15 mW of optical power delivered toward the probe, with 13.5 mW reaching the sample.

The common-path probe, shown schematically in Figure 1b, consists of a single-mode optical fiber (SMF-28) placed inside a ferrule, a UV-curable index-matching adhesive NOA 141, Norland Optical Adhesive (Norland Products, Inc., Jamesburg, NJ, USA), a free-space region, and a 2.5 mm diameter BK7 ball lens mounted inside a transparent tube. A small amount of adhesive was initially filled inside the ferrule capillary in front of the optical fiber tip. The fiber was then retracted to positions ranging from 0 to 500 µm in 50 µm increments using a motorized translation stage (LNR502/M, Thorlabs, Newton, NJ, USA), which provided a positioning resolution of 0.1 µm. The space between the fiber end-face and the adhesive–air interface was filled with the same adhesive, such that the selected fiber displacement defined both the fiber-to-reflector separation and the adhesive optical path length. The adhesive was then cured at this position, forming a fixed adhesive–air interface that provided the partial Fresnel reflection for the reference signal. Probes with different fiber-to-reflector separations were fabricated, and the corresponding reference power was measured after curing. The fiber-to-reflector separation was fixed during subsequent OCT imaging.

The adhesive has a refractive index of 1.458, which is closely matched to that of the optical fiber, thereby minimizing Fresnel reflection at the fiber–adhesive interface. In contrast, the adhesive–air interface provides the partial Fresnel reflection used as the reference signal. Following this interface, a free-space region of approximately 2 mm separates the reflector from the ball lens, focusing the light approximately 4 mm away from the ball lens. This fiber-tip-to-lens distance can be adjusted to modify the focal position according to the requirements of the application. The probe design provides approximately 2 mm of available space between the adhesive–air interface and the ball lens, allowing the fiber position to be varied within this range while maintaining the compact probe configuration. The ball lens was selected as a compact and cost-effective focusing optics. The reference and sample fields share approximately the same downstream optical path through the probe, providing the common-path configuration and reducing sensitivity to environmental disturbances.

Before applying the adhesive, the Fresnel-reflected optical power from the fiber–air interface was approximately 480 µW. After fabrication, the reference power corresponding to each fiber-to-reflector separation was measured and used for the subsequent experiments.

The resulting interference signal was returned through the optical circulator and detected using a photodetector (PDB465C, Thorlabs, Newton, NJ, USA). The detected interference signal was digitized using a high-speed data acquisition card (SA220P, Acqiris SA, Geneva, Switzerland) at a sampling rate of 2 GS/s, with 8196 samples acquired per A-line at a swept-source repetition rate of 100 kHz. A reference spectrum was acquired at the beginning of the experiment and subsequently subtracted to minimize fixed-pattern artifacts in the images arising from multiple reflections within the probe. The background-subtracted spectrum was then Fourier transformed to obtain the axial profile of the sample. System control, data acquisition, and signal processing were implemented using custom software developed in LabVIEW (National Instruments, Austin, TX, USA), including laser synchronization, digitizer configuration, data acquisition, and OCT image reconstruction.

### 2.2. Numerical Modeling of the Common-Path Probe

The numerical model was developed in Zemax OpticStudio to evaluate the effect of fiber-to-reflector distance on the reference power. The optical fiber was modeled with a numerical aperture (NA) of 0.14, and the UV-curable adhesive was modeled with a refractive index of 1.458. The ball lens was not included in the numerical model because it was used experimentally only to focus the light onto the sample and was not required for evaluating the fiber-to-reflector coupling and reference signal.

Experimentally, the fiber was retracted relative to the adhesive reflector using a motorized translation stage. Light emerging from the fiber propagated to the reflector and returned to the fiber, resulting in a round-trip propagation path. In the Zemax model, this optical coupling was represented using a fiber-to-fiber configuration. The resulting coupled optical power was used to evaluate the reference power as a function of the fiber-to-reflector distance. The complete Zemax OpticStudio files and detailed simulation parameters are provided as Supplementary Information.

## 3. Results

The performance of the SS-OCT system was experimentally characterized in terms of its axial resolution, spatial resolution, and sensitivity roll-off. The measured axial and lateral resolutions were approximately 9 µm and 8 µm, respectively. The 3 dB system sensitivity roll-off was measured to be approximately 4 mm. The overall imaging depth of the system was measured to be 35 mm.

### 3.1. Reference Power Optimization Through Simulation and Experimental Validation

**Figure 2:**
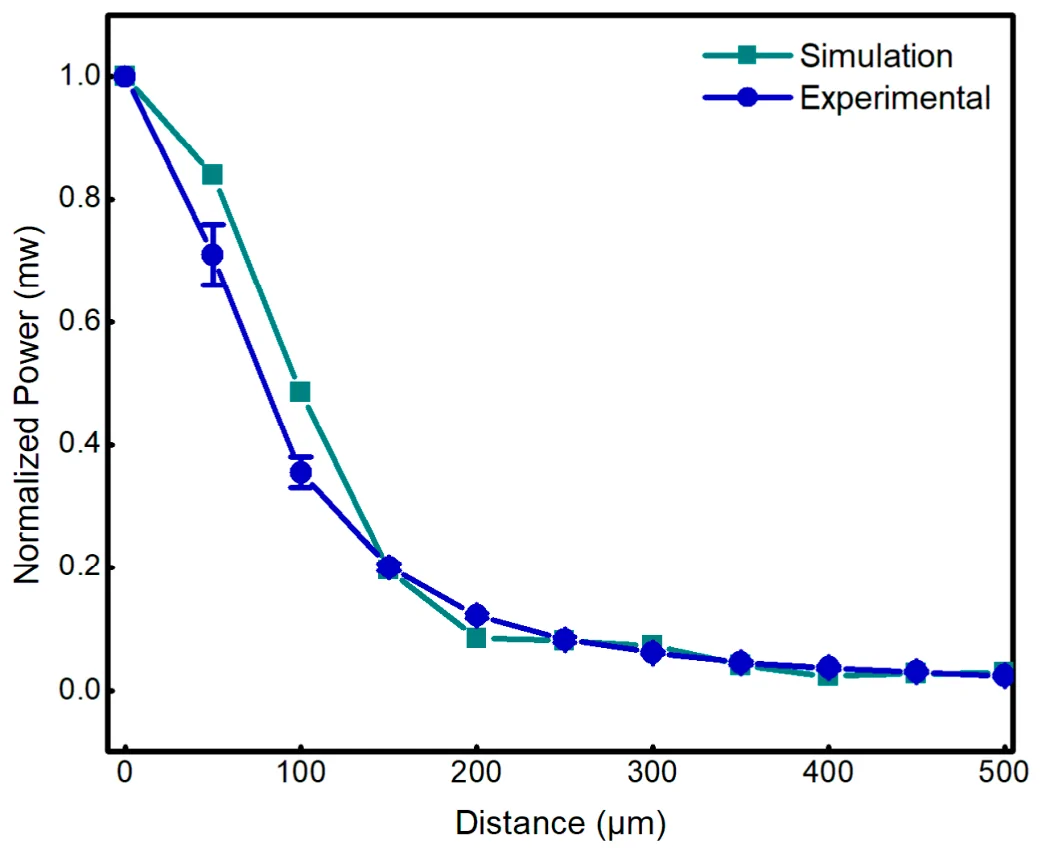
Normalized reference power versus fiber-to-reflector distance in a CP-OCT probe, comparing numerical simulation and experimental measurements. Error bars represent the standard deviation of the normalized reference power measured across 10 fabricated probes.

The reference power of the CP-OCT probe was optimized through numerical simulation and experimental validation, as shown in Figure 2. In a common-path configuration, the reference and sample signals propagate along the same optical path; therefore, the generated reference power is strongly influenced by the probe geometry, particularly the separation between the fiber end-face and the reflective interface. Optimizing this distance is essential for achieving sufficient interference signal strength and maintaining high imaging sensitivity.

The effect of fiber-to-reflector separation on optical coupling efficiency was first investigated using Zemax OpticStudio. The reference power coupling was modeled using the fiber coupling analysis, where the fiber-to-reflector distance was varied from 0 to 500 µm with a 50 µm step size. The coupled optical power was calculated at each position and normalized to the maximum value obtained at zero separation.

Experimental measurements were subsequently performed using the fabricated CP-OCT probe. The probe was fabricated by using a single-mode fiber and adding an index-matched UV-curing epoxy in front of the fiber. The epoxy–air interface generated the reference signal through partial Fresnel reflection, which was coupled back to the core of the fiber. The fiber core position relative to the reflector interface was adjusted in controlled increments, and the returned optical power was measured over the same displacement range used in the simulation. The measured values were normalized to the maximum power and compared directly with the simulated results.

The normalized reference power as a function of the distance between the fiber core and the epoxy–air interface is shown in Figure 2. Both simulation and experimental results exhibit a similar decreasing trend with increasing distance. The reduction in coupled power is attributed to the reduced overlap between the returning optical field and the guided fiber mode as the distance increases. The most significant power variation occurs at smaller distances, demonstrating the sensitivity of the reference signal to probe geometry.

The close agreement between simulation and experimental measurements confirms the validity of the optical model and highlights the importance of controlling fiber-to-reflector spacing during probe fabrication. Since OCT sensitivity depends on the balance between reference and sample signals, insufficient reference power reduces interference fringe visibility and SNR, whereas excessive reference power can reduce detector dynamic range and increase noise contributions. Therefore, optimizing the fiber-to-reflector separation provides an effective approach for achieving balanced reference power and improved OCT imaging performance. To assess reproducibility, measurements were performed using 10 fabricated probes. Error bars in Figure 2 represent the standard deviation of the normalized reference power, indicating probe-to-probe variation.

### 3.2. Effect of Reference Power on Signal-to-Noise Ratio and Phase Stability

**Figure 3:**
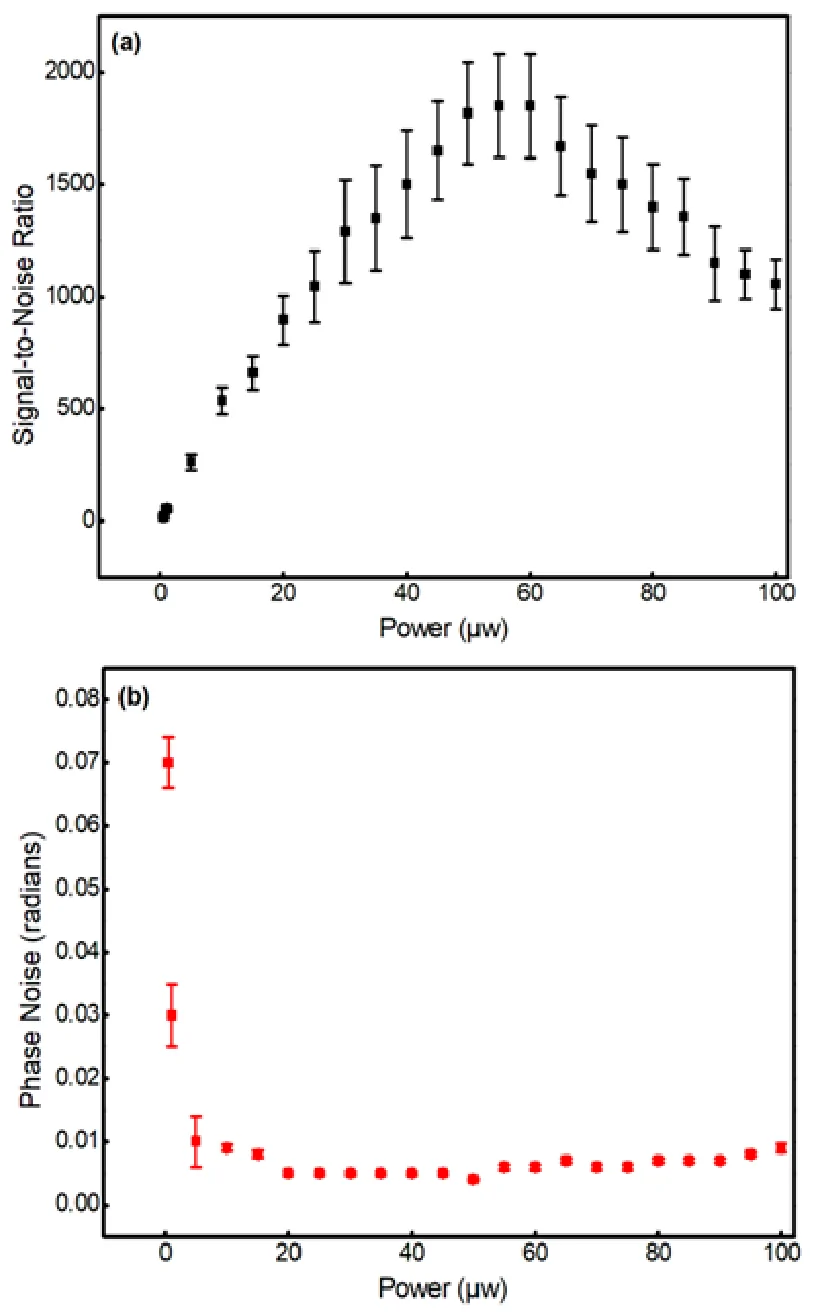
Measured SNR and phase noise as a function of reference optical power. (**a**) SNR increases with reference power, reaches an optimum in the approximately 40–60 µW range, and decreases at higher power levels. (**b**) Phase standard deviation decreases with increasing reference power and remains relatively stable above approximately 20 µW.

The influence of reference power on the SNR and phase stability of the CP-OCT system was investigated and is shown in Figure 3. To characterize the system SNR and phase stability, multiple probes with different reference power levels were fabricated. A mirror was positioned in front of the common-path probes, and the optical power coupled back into the probe from the mirror was maintained at 1 µW for all measurements. The sample-return power of 1 µW was selected to provide a representative weak-signal level corresponding to the optical power expected from tissue backscattering in our OCT measurements. The SNR of the amplitude of the A-scan was calculated as the ratio of the peak amplitude of the Fast Fourier Transform (FFT) spectrum to the standard deviation of the noise floor. Phase stability was quantified by calculating the standard deviation of the measured phase over the acquired measurements. At low reference-power levels, the system exhibited reduced SNR and increased phase fluctuations due to the insufficient reference field strength. The weak interference signal generated under these conditions limits fringe visibility and increases the influence of noise on both the detected signal and phase measurements.

With increasing reference power, a significant improvement in the SNR and the phase stability was observed. The enhanced reference field strengthens the interference signal, resulting in reduced phase variations. The phase noise decreased with increased reference power and remained nearly constant above approximately 20 µW, as shown in Figure 3b, suggesting that further increases in reference power provided limited improvement and that the residual phase noise was likely influenced by system-level noise sources.

Further increases in reference power resulted in diminishing improvements in SNR, with the optimum range observed at approximately 20–60 µW. Beyond this range, the SNR gradually decreased, potentially due to reduced detector dynamic-range margin and increased intensity-related noise. Although increasing the reference power improved the measured SNR (Figure 3a), the SNR error bars initially increased with reference power before decreasing as the detector approached saturation. Since the FFT peak amplitude remained relatively stable, the increased SNR variability was primarily associated with fluctuations in the noise-floor standard deviation. At intermediate reference power, increased optical power can enhance power-dependent noise sources, such as shot noise and relative intensity noise, leading to greater variability of the noise floor. At higher power, the reduction in error bars coincided with the detector approaching saturation and is therefore likely associated with its nonlinear response rather than a reduction in intrinsic noise.

According to the manufacturer’s specifications, the detector used in this study has a saturation level of approximately 120 µw under continuous illumination. However, in practice, due to the modulation of the interference signal, the detector approached saturation at a reference power of approximately 60 µw.

These results demonstrate that reference power optimization is essential in CP-OCT systems. An appropriate reference power level provides a balance between interference modulation, SNR, and phase stability, while excessive reference power does not contribute to further phase improvement and may degrade overall signal quality.

### 3.3. Effect of Reference Power on Image Quality in CP-OCT Systems

**Figure 4:**
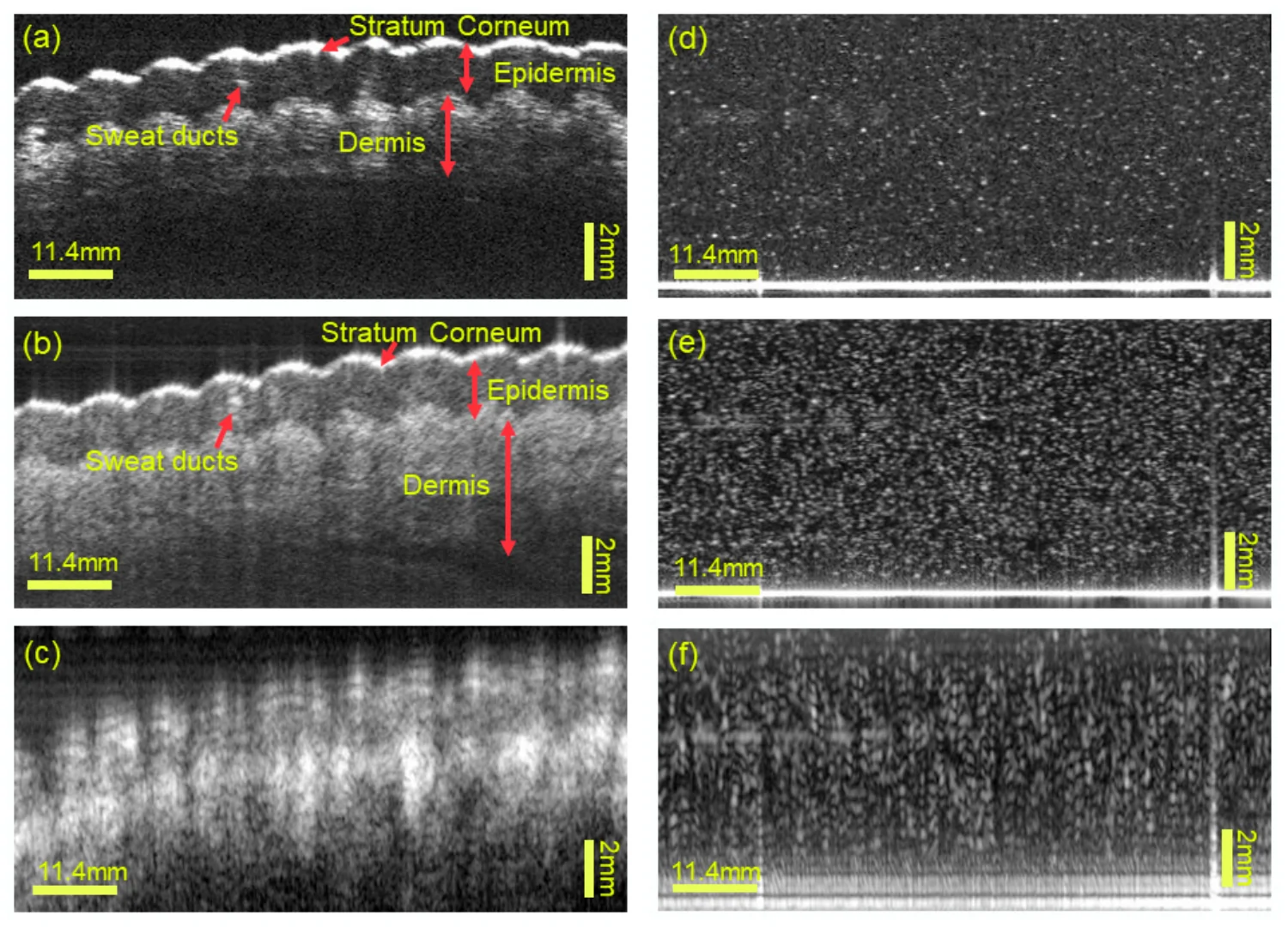
OCT images of a human fingertip: (**a**) at 1 µW, (**b**) at 55 µW, and (**c**) at 110 µW, and an OCT phantom: (**d**) at 1 µW, (**e**) at 55 µW, and (**f**) at 110 µW. The images illustrate the effect of reference power on OCT image quality.

The influence of reference power on OCT image quality was evaluated using both an in vivo human fingertip and an OCT imaging phantom at reference power levels of 1 µW (low reference power), 55 µW (optimal reference power), and 110 µW (high reference power). Representative OCT images are shown in Figure 4. A motorized scanning stage was used to scan the beam across the sample.

At a reference power of 1 µW, as shown in Figure 4a, the acquired images exhibited relatively low signal intensity and reduced contrast due to the limited interference signal. In the fingertip images, only prominent superficial features, including the stratum corneum and sweat ducts, were distinguishable, while the deeper tissue layers were poorly resolved. Similarly, the OCT phantom image showed only a limited number of scattering features with reduced visibility, as shown in Figure 4d.

Increasing the reference power to 55 µW, as shown in Figure 4b, produced a marked improvement in image quality. The higher SNR resulted in improved image quality and detectability of structural features. In the fingertip images, the layered architecture of the skin, including the stratum corneum, epidermis, dermis, and sweat ducts, was clearly visualized with well-defined tissue boundaries. The OCT phantom, as shown in Figure 4e, also exhibited improved visibility of the embedded scattering features, with a greater number of nanoparticles clearly resolved throughout the imaging depth.

When the reference power was further increased to 110 µW, as shown in Figure 4c, image quality deteriorated despite the higher optical power, potentially due to the reduced detector dynamic range at higher reference levels. Consequently, the structural features became less distinguishable, resulting in poorer delineation of the skin layers in the fingertip images. A similar trend was observed in the OCT phantom, shown in Figure 4f, where the scattering features became less distinct, resulting in reduced image quality.

For quantitative image analysis, signal ROIs (50 × 50 µm^2^) were selected within the epidermis and dermis, while a signal-free region above the stratum corneum was used for background estimation. The same ROI-selection approach was maintained across the different reference-power conditions to ensure consistent comparison. For the OCT phantom, the maximum intensity of an individual nanoparticle peak was used as the signal.

These results demonstrate that OCT image quality is highly dependent on the reference power. While insufficient reference power limits signal strength and image contrast, excessive reference power may reduce the available detector dynamic range and degrade structural information. An optimal reference power of approximately 55 µW provided the best balance between signal intensity and detector dynamic range, resulting in improved detection of biological tissue layers and phantom structures.

To quantitatively evaluate the effect of reference power on image quality, as shown in Figure 4, the SNR was calculated as the ratio of the mean signal intensity to the standard deviation of the background intensity. SNR was evaluated separately for the epidermis, dermis and OCT phantom at reference powers of 1, 55, and 110 µW. The results are summarized in Table 1.

## 4. Discussion

This study shows that the performance of a CP-OCT probe is heavily influenced by the reference power generated within it. By combining numerical simulations with experimental validation, the relationship between fiber-to-reflector distance, reference power, phase stability, SNR, and image quality was systematically examined. The findings indicate that adjusting the probe geometry offers an effective way to optimize the reference signal without adding extra optical components or increasing system complexity.

The numerical simulation accurately predicted the experimentally observed variation in reference power with fiber-to-reflector separation, confirming that the reduction in coupled power is primarily governed by beam divergence and decreasing mode overlap. The close agreement between simulation and experiment indicates that optical modeling can serve as a reliable design tool for selecting the probe geometry before fabrication, thereby reducing development time and minimizing trial-and-error optimization.

The experimental results further demonstrate that reference power has a direct influence on OCT performance. At low reference power, the interference signal is weak, resulting in reduced SNR, poorer phase stability, and limited visualization of tissue structures. Increasing the reference power improves the interference signal, leading to enhanced SNR, reduced phase fluctuations, and improved delineation of the stratum corneum, epidermis, dermis, and sweat ducts in fingertip images. Similar improvements were observed in the OCT phantom, where scattering features became more clearly resolved at the optimized reference power.

However, increasing the reference power beyond the optimal range did not further improve image quality. While insufficient reference power limits signal strength and image contrast, excessive reference power may reduce the available detector dynamic range and degrade structural information. These findings demonstrate that maximizing reference power does not necessarily maximize imaging performance. Rather, an optimum reference power must be selected to balance the interference signal with the limitations imposed by the detector and system noise.

Unlike conventional Michelson-based OCT systems, where the reference power can be adjusted independently using a variable attenuator, the reference power in common-path OCT is intrinsically determined by the probe design. Consequently, optimization of the fiber-to-reflector spacing represents a practical and effective strategy for controlling the reference signal at the probe level. This approach simplifies probe fabrication while maintaining stable and reproducible imaging performance.

The optimized common-path probe has significant potential for biomedical imaging applications. In biomedical imaging, compact common-path probes are well suited for endoscopic OCT, intravascular imaging, and dermatology, where mechanical stability and miniaturization are essential. Improved reference power optimization can enhance image quality and phase stability, facilitating more reliable visualization of tissue microstructures and supporting quantitative OCT techniques such as angiography and elastography.

In practical biological OCT imaging, the optical power returned from the sample is typically substantially lower than the reference power. In a conventional OCT system, the reference power is typically optimized during system setup and maintained during image acquisition, whereas the sample-return power varies depending on tissue reflectivity, scattering, and local tissue structure. The same principle applies to the proposed common-path probe. Because the reference reflector is incorporated into the probe during fabrication, the reference power is fixed for a given probe and cannot be dynamically adjusted during imaging. Therefore, the reference power should be selected during probe fabrication to provide an appropriate operating condition for the intended imaging application.

Although the present study establishes practical guidelines for reference power optimization, several aspects warrant further investigation. Future work could extend the numerical model to include additional optical interfaces and reflector materials, evaluate different probe geometries, and investigate the influence of optimized reference power on advanced functional OCT modalities. These studies would further improve probe performance and broaden the applicability of compact common-path OCT systems.

The common-path probe demonstrated in this study represents a proof-of-concept optical configuration. For future endoscopic applications, the probe could be integrated with a rotary scanning mechanism to enable side-viewing imaging while maintaining its compact optical configuration.

Long-term stability and reproducibility of the UV-cured reflector are important considerations for practical CP-OCT probes. UV-curable optical adhesives can exhibit dimensional and mechanical changes with temperature and long-term aging. In the present study, these effects were not systematically evaluated. Long-term stability should be further evaluated through repeated measurements under controlled temperature and mechanical disturbances. These measures would help minimize probe-to-probe variation and maintain the reference power within the desired operating range.

The potential effect of the additional BK7 ball lens in the reference arm on the axial point-spread function (PSF) should also be considered. In our study, the OCT system operates at a center wavelength of approximately 1310 nm with a spectral bandwidth of 105 nm. Because the 2.5 mm thick ball lens is traversed twice in the reference arm, the effective dispersive path length is approximately 5 mm. At this wavelength, BK7 glass exhibits relatively low group-velocity dispersion (2.1 fs^2^/mm), resulting in only a small accumulated dispersion (10.5 fs^2^) over this additional optical path. The corresponding dispersion-induced broadening of the axial PSF is therefore expected to be negligible (roughly 0.1%) compared with the intrinsic axial resolution of the system. Thus, the presence of the additional 2.5 mm thick ball lens in the reference arm is not expected to have a significant effect on the measured axial resolution.

Data security is also an important consideration for clinical OCT imaging, particularly when images are stored or transmitted through networked systems. Recent image-encryption approaches based on cross-image permutation and diffusion have demonstrated effective protection of multiple images against statistical and differential attacks. Such encryption strategies could be incorporated into future CP-OCT imaging systems to enhance the privacy and secure transmission of patient-derived OCT images [30].

Overall, this work provides a practical framework for optimizing reference power in CP-OCT probes through the combined use of optical simulation and experimental validation. The findings establish design guidelines for selecting an appropriate fiber-to-reflector spacing to achieve balanced reference power, thereby improving SNR, phase stability, and image quality while preserving the simplicity and robustness of the common-path probe architecture.

## Figures and Tables

**Table 1:** Quantitative SNR evaluation of the epidermal and dermal regions at different reference powers.

Reference Power (µw)	Epidermis (SNR)	Dermis (SNR)	OCT Phantom (SNR)
1	87.23	93.61	96
55	113.9	120.93	115
110	89.36	100	98

## Data Availability

The original contributions presented in this study are included in the article and Supplementary Material. Further inquiries can be directed to the corresponding author.

## Supplementary Materials

The following supporting information can be downloaded at: https://www.mdpi.com/article/10.3390/jimaging12090454/s1, Figure S1: Representative reference spectra at different reference-power levels, showing detector saturation at 110 μW. Positive and negative signal values result from the applied voltage offset; Table S1: Zemax simulation results for the silica single-mode fiber with a numerical aperture (NA) of 0.14.
