# Supplementary material for: Reference Power Optimization for Common-Path Probes in Optical Coherence Tomography
Source: J Imaging. 2026 Sep 20;12(9):454. doi: 10.3390/jimaging12090454 (PMC13608142; doi:10.3390/jimaging12090454)
Supplement: Supplementary file 1 [file jimaging-12-00454-s001.zip › jimaging-4476087-supplementary.pdf]

Journal of Imaging

Supplementary Material

Reference Power Optimization for Common-Path Probes in Optical Coherence Tomography

Asha Parmar *, Sora Alghziwatalkhawaldh, Shantanu Chauhan and Kanwarpal Singh

Department of Electrical and Computer Engineering, McMaster University, Hamilton, ON L8S 4L7, Canada; alghziws@mcmaster.ca (S.A.); shant8@mcmaster.ca (S.C.); singk33@mcmaster.ca (K.S.)
* Correspondence: ashaa3@mcmaster.ca

The detector response was recorded at different reference powers (1 µW, 55 µW, and 110 µW) to evaluate the effect of increasing optical reference power on the detector output. With increasing reference power, the Intensity of the detected spectrum increases until the detector approaches its dynamic range limit. At 110 µW, the spectrum exhibits clear clipping and distortion at the upper and lower signal limits, indicating detector saturation and providing evidence for the degradation in OCT image quality observed at this power level. These negative values represent the offset-adjusted electrical detector signal and do not correspond to negative optical power.

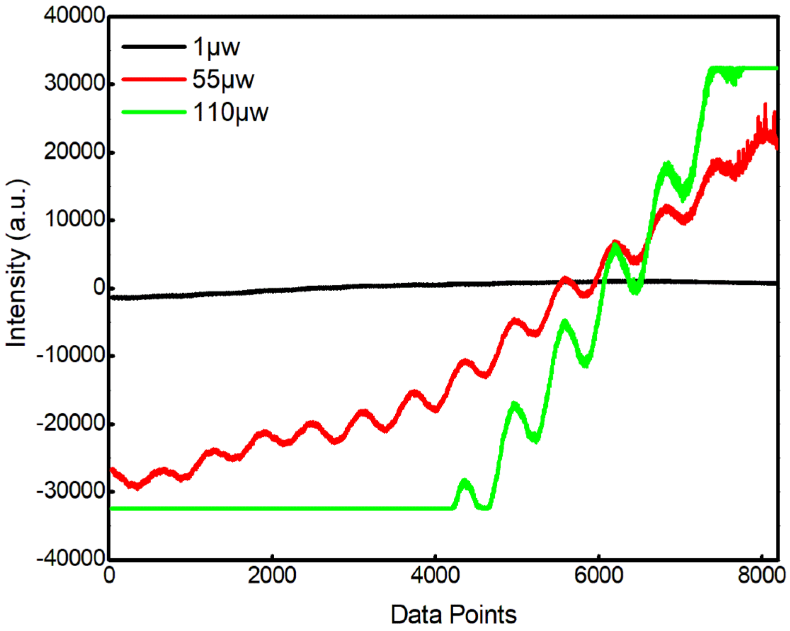

Figure S1. Representative reference spectra at different reference-power levels, showing detector saturation at 110 µW. Positive and negative signal values result from the applied voltage offset.

Table S1. Zemax simulation results for the silica single-mode fiber with a numerical aperture (NA) of 0.14.

Distance (µm)	System Efficiency	Receiver Efficiency	Coupling Efficiency
0	0.864389	0.857048	0.740823
50	0.864389	0.718533	0.621092
100	0.864389	0.416759	0.360242
150	0.864389	0.167517	0.144800
200	0.864389	0.072292	0.062489

250	0.864389	0.068653	0.059343
300	0.864389	0.062063	0.053646
350	0.864389	0.035360	0.030565
400	0.864389	0.020222	0.017480
450	0.864389	0.023959	0.020710
500	0.864389	0.025542	0.022079
